# A Simple Method for Estimation of the Scattering Exponent of Nanostructured Glasses

**DOI:** 10.3390/ma16072630

**Published:** 2023-03-26

**Authors:** Michael Shepilov, Olga Dymshits, Aleksandr Zhilin

**Affiliations:** 1 Department of Glass, Vavilov State Optical Institute, 36 Babushkina St., 192171 Saint Petersburg, Russia; m.shep@mail.ru; 2 D.V. Efremov Institute of Electrophysical Apparatus, Metallostroy, Doroga na Metallostroy, 3 Bld., 196641 Saint Petersburg, Russia; zhilin1311@yandex.ru

**Keywords:** nanostructured glasses, glass-ceramics, phase-separated glasses, optical spectra, scattering coefficient, anomalous light scattering, scattering exponent

## Abstract

For most of nanostructured glasses (NGs) (phase-separated glasses and glass-ceramics), the light scattering coefficient (turbidity) is described by a power function of the inverse wavelength with an exponent which differs appreciably from the Rayleigh value 4 and is called the scattering exponent. The knowledge of the scattering exponent of a material is important from both fundamental and practical points of view. Previously, we developed three rather complex methods to determine the scattering exponent. Here, we present a novel simple express method for its estimation. In the method, the measured optical density for only one sample is used, the refractive index of the material is not required, and the dispersion of refractive index is assumed to be insignificant. The method is based on the differentiation of the measured optical density with respect to the wavelength. The scattering exponent values obtained by the new method for NGs of different types are in good agreement with those found by the traditional methods. The new method is found to be applicable even to NGs with high dispersion of refractive index. Thus, the new method does not require the data on the refractive index dispersion and can be applied without restrictions.

## 1. Introduction

Quite often, the scattering coefficient (turbidity) $\alpha_{s}$ of nanostructured glasses (NGs) (phase-separated glasses and glass-ceramics) is described by the power function of the inverse wavelength in a certain wavelength range from $\lambda_{\min}$ to $\lambda_{\max}$ [1,2]

(1) $$\alpha_{s}(\lambda )≅a{\left(1/\lambda \right)}^{s},\;(a,s=\mathrm{constant},\;\lambda_{\min}<\lambda <\lambda_{\max}),$$

where λ is the wavelength of light in vacuum and the value of *s* is appreciably greater than the Rayleigh value 4 ([3], Chap. 5, p. 132). We will call *s* the scattering exponent.

This phenomenon called anomalous light scattering is caused by the interference of light scattered by different elements of the structure of NGs [1]. Because of interference, the scattered light is directed predominantly into the backward hemisphere [1] (also see Figures 3, 4, and 6–8 in Ref. [4]), whereas Rayleigh scattering is characterized by forward–backward symmetry ([3], Chap. 5, p. 134). In addition, the interference suppresses light scattering, so that the measured scattering coefficient of NG is significantly less than the scattering coefficient calculated for independent Rayleigh scatterers (for example, see Figure 18 in Ref. [5]).

The anomalous light scattering by phase-separated glasses has been studied since the mid-1950s [6,7,8,9] and was summarized in a review [1]. However, this phenomenon is ignored in reviews [10,11], books [12,13], and papers (for example, see Refs. [14,15]) devoted to glass-ceramics, in which only the $\lambda^{-4}$ dependence of the scattering coefficient is considered.

It should be noted that the $\lambda^{-4}$ dependence takes place not only for independent scattering by small particles (Rayleigh scattering), but also for the scattering of a system described by a simple correlation function with small correlation length in the Debye and Bueche approach [16] (for details, see Ref. [17]). In Refs. [11,12,13], one more model is mentioned, in which the $\lambda^{-4}$ dependence is predicted. The model was proposed by Hopper [18]. He considered the scattering of light by a spinodal structure consisting of two phases with sharp interfaces and equal volume fractions and demonstrated that interference of scattered light may lead to significant suppression of scattering. It is noteworthy that Equation (70) of Ref. [18] obtained for turbidity of the interconnected phases is sometimes discussed in terms of particles [11,12,13].

Expecting the $\lambda^{-4}$ dependence, the authors of experimental works often present the internal optical density (or similar quantity) as a function of $\lambda^{-4}$ in the hope of obtaining a straight line. In most cases, the obtained line is a curve that does not have a linear section. Nevertheless, the authors limit themselves to a linear approximation of the curves, while taking into account the nonlinearity and determining the scattering exponent provide additional information about the material, at least about the spectral dependence of its transparency. As examples, the glass-ceramic J from Ref. [14] and the nepheline glass-ceramic from Ref. [15] may be mentioned, for which a rigorous analysis resulted in the scattering exponent values of 5.7 [2] and 5.0 [17], respectively, instead of 4.

An analysis of the spectra of a number of glass-ceramics presented in the literature led us to the conclusion that glass-ceramics are usually characterized by anomalous light scattering [2].

There are a number of models for the explanation of anomalous light scattering, in which the processes of NGs formation are taken into account.

At an early stage of diffusion-limited growth of particles (crystals) in glass, their diffusion zones grow independently. As shown in Refs. [19,20], the interference of light scattered by a particle and its diffusion zone leads to suppression of the dipole component in scattering and to anomalous light scattering with the scattering exponent s=8. This model is inapplicable if diffusion zones impinge on one another, which happens at the end of the stage of active diffusion growth.

Hendy [21] used the simulated literature data on the structure factor of a system undergoing late-stage spinodal decomposition. He averaged these literature data and theoretically predicted anomalous light scattering in this system in the long-wavelength spectral range with the scattering exponent s=8. To describe anomalous light scattering by glass-ceramics, he also assumed that the spinodal and binodal structures are similar at the Ostwald ripening stage and applied his theory to glass-ceramics formed by the binodal mechanism.

The authors of Ref. [22] considered the interference of light scattered by an NG in the course of the diffusional growth of particles and concluded that the interference reduces turbidity significantly below that of independent Rayleigh scatterers if the wavelength is sufficiently long. For the 3D case, a rough approximation for the structure factor of the system of monodisperse particles was used in the theoretical consideration, which leads to the value s=6 of the scattering exponent.

In Refs. [23,24], the model NG was considered formed towards the end of the stage of active diffusional growth of simultaneously nucleated particles. Its structure and light scattering were simulated using a simple geometrical probability model proposed earlier. The simulation carried out in the model demonstrates the formation of a system of polydisperse particles with correlated arrangement whose scattering is not independent and exhibits strong interparticle interference if the wavelength of light is sufficiently long. The model demonstrates all effects of anomalous light scattering and leads to the value s=7.1 of the scattering exponent.

The particles in the model [23,24] are assumed to be spherical, and the results of simulation can be strictly applied only to some special cases, such as, for example, the glass G2 (Section 2.2.1) with spherical particles [25]. The experimental value of the scattering exponent for this glass ([26] and Section 2.2.1 of the present paper, Equations (15) and (16)), s≅6.9, is close to simulated one, s=7.1.

In general, nanocrystals in glass-ceramics have a complex shape and differ from one another. Correspondingly, the problem of the theoretical description of the structure and light scattering of such material is very difficult and is waiting for a solution.

If we speak about the theoretical description of anomalous light scattering in NGs, Refs. [4,27] should be mentioned where the relationship between the spectral dependence of the scattering coefficient (Equation (1)) and the angular distribution of the scattered light was examined theoretically.

It should be noted that in some cases, the deviation of the scattering exponent from the Rayleigh value of 4 is not related to the interference effects. For NG, it may be caused by the dispersion of the refractive indices of particles (crystals) and the matrix [5,28]. For example, the values s=4.16−4.17 were demonstrated in Ref. [5] (see p. 166 and footnote 4 in this reference), and an even larger value of s=4.8 was reported in Ref. [28]. Sometimes, values of the scattering exponent less than 4 were also observed (see, e.g., Ref. [29]). Such scattering may be ascribed to scattering by large particles (the Mie scattering) [30]. For example, the scattering with the scattering exponent s=2 can be observed for optically soft spherical particles (see Figure 1 in Ref. [17]). In addition, the value of s=2 may be inherent in transparent ceramics (see section III(4) of Ref. [31], Equation (7) in Ref. [32], or Equations (5a), (13), and (18) in Ref. [33]).

The scattering exponent determines the spectral dependence of scattering and the transparency of the material. This information is important for the optical applications of NGs. On the other hand, the value of the scattering exponent is related to the scattering mechanism and its knowledge may help to formulate certain assumptions about the structure of the material. Thus, the experimental and theoretical study of anomalous light scattering is important from both fundamental and practical points of view.

To reveal anomalous light scattering in NGs, one can analyze the behavior of the light extinction coefficient α(λ) in the spectral range, where the absorption is negligible and the extinction coefficient is equal to the scattering coefficient:

(2) $$\alpha \left(\lambda \right)=\alpha_{s}(\lambda )$$

The extinction coefficient of a material may be found using spectrophotometers in transmission experiments with a normal incidence of light on a plane-parallel sample ([3], Section 2.8). Here, it should be noted that in the case of light-scattering material, the estimation of the extinction coefficient in such experiments is complicated by unwanted contributions by the scattered light to the readings of the detector of a spectrophotometer. However, it was shown [34] that this contribution is negligible if the acceptance angle of the detector is about 6° (as for the serial spectrophotometer Shimadzu UV 3600) and the internal optical density D(λ) of a sample of nanostructured light-scattering material with refractive index n≳1.5 satisfies the condition:

(3) D(λ)≲2.

This condition should be kept in mind during the preparation of scattering samples and the measurement of their optical density.

The extinction coefficient α(λ) is directly related to the internal optical density D(λ) of the sample of known thickness h [2,34] by the relation:

(4) $$\alpha \left(\lambda \right)=\left[D\left(\lambda \right)\ln10\right]/h.$$

To obtain the internal optical density D(λ) from transmission experiments, one should exclude the optical density of reflection losses, $D_{\mathrm{refl}}(\lambda )$, from the measured value of optical density, $D_{m}(\lambda )$. **The first (direct) method** to achieve this is to measure the optical densities ($D_{1m}\left(\lambda \right)$, $D_{2m}(\lambda )$) of two samples of different thicknesses ($h_{1}$, $h_{2}$) and use the equation (see, for example, Equation (3) in Ref. [34]):

(5) $$\alpha \left(\lambda \right)=\{\left[D_{2m}\left(\lambda \right)-D_{1m}\left(\lambda \right)\right]\;\ln10\}/(h_{2}-h_{1}).$$

**The second method** to determine the internal optical density D(λ) of the sample is to calculate the reflection losses using the measured value of the refractive index n(λ) of the material and to subtract the optical density $D_{\mathrm{refl}}(\lambda )$ of reflection losses from the measured optical density $D_{m}(\lambda )$ of the sample (e.g., see Equations (15)–(18) in Ref. [34]):

(6) $${D\left(\lambda \right)=D_{m}\left(\lambda \right)-D}_{\mathrm{refl}}\left(\lambda \right),\;D_{\mathrm{refl}}\left(\lambda \right)≅-2\log_{10}\{4n(\lambda )/{\left[n\left(\lambda \right)+1\right]}^{2}\}.$$

For the strict application of the second method, a dispersion of the refractive index should be taken into account (see, e.g., Ref. [35]). If the value of the refractive index is known only for one wavelength, we have to assume that the dispersion is small and apply the second method approximately.

**The third method** can be applied if the variation of the refractive index of the material during phase transformation (phase separation and crystallization) is insignificant. This means that the optical density $D_{\mathrm{refl}}(\lambda )$ of reflection losses does not change in the course of phase transformation. In this case, one can measure the optical density $D_{\mathrm{ig},m}(\lambda )$ of a sample of the initial glass and the optical density $D_{\mathrm{htg},m}(\lambda )$ of this sample after its heat treatment. These optical densities can be presented as

(7) $$D_{\mathrm{ig},m}\left(\lambda \right)=D_{\mathrm{refl}}\left(\lambda \right)+D_{\mathrm{ig}}\left(\lambda \right),\;D_{\mathrm{htg},m}\left(\lambda \right)=D_{\mathrm{refl}}\left(\lambda \right)+D_{\mathrm{htg}}\left(\lambda \right),$$

where $D_{\mathrm{ig}}(\lambda )$ and $D_{\mathrm{htg}}(\lambda )$ are internal optical densities of the sample before and after heat treatment, respectively. Thus, the difference in measured optical densities of the sample after and before heat treatment:

(8) $$∆D\left(\lambda \right)=D_{\mathrm{htg},m}\left(\lambda \right)-D_{\mathrm{ig},m}\left(\lambda \right)=D_{\mathrm{htg}}\left(\lambda \right)-D_{\mathrm{ig}}\left(\lambda \right),$$

gives the variation of the internal optical density of the sample caused by phase transformations. This variation is related to variation in absorption and scattering in the material due to structural changes induced by phase transformations. The corresponding variation in the extinction coefficient is expressed as:

(9) $$\Delta \alpha \left(\lambda \right)=[\Delta D\left(\lambda \right)\;\ln10]/h,$$

where the thickness h is practically the same for the initial and heat-treated sample.

To our knowledge, the third method of exclusion of reflection losses from the measured optical density was first proposed in Ref. [36] (see Equation (3) of this reference). The method allowed us to observe the small changes in the shape of Er^3+^ ion absorption peaks and the appearance of light scattering due to the precipitation of ZnO nanocrystals, and to determine the values of the scattering exponent. This method seems to be the simplest of the three.

Thus, the application of any of the three methods presented above gives us the opportunity to determine the wavelength dependence of the scattering coefficient in the spectral range where absorption is negligible, to analyze it and, if this dependence obeys Equation (1), to find the value of the scattering exponent, s.

In this paper, we present a novel simple express method for the estimation of the scattering exponent. In the method, the measured optical density $D_{m}(\lambda )$ for only one sample is used, the refractive index of the material is not required, and the dispersion of the refractive index is assumed to be insignificant. In the course of our study, it was found that the method is also applicable to the cases of materials with high dispersion of the refractive index.

## 2. A Simple Method for Estimation of the Scattering Exponent and Its Testing

### 2.1. The Method

Let us assume that Equation (1) is satisfied in some spectral range, where absorption is negligible, and that the optical density of reflection losses in this range is independent of wavelength:

(10) $$D_{\mathrm{refl}}\left(\lambda \right)=D_{\mathrm{refl}}=\mathrm{constant}.$$

Then, using Equations (1), (2), (4), (6), and (10), one can express the measured optical density as

(11) $${D_{m}\left(\lambda \right)=D\left(\lambda \right)+D}_{\mathrm{refl}}=b{\left(1/\lambda \right)}^{s}+D_{\mathrm{refl}},\;(b=ah/\ln10=\mathrm{constant}).$$

Differentiation of this equation gives the relation:

(12) $$dD_{m}(\lambda )/d\lambda =-bs\lambda^{-s-1},$$

which may be rewritten as:

(13) $$\log_{10}(-dD_{m}(\lambda )/d\lambda )=-\left(s+1\right)\log_{10}\left(\lambda \right)+c,\;(c=\log_{10}(bs)=\mathrm{constant}).$$

This means that the dependence of $\left(-dD_{m}(\lambda )/d\lambda \right)$ on λ presented as a log–log plot will give a straight line with a slope $-\left(s+1\right)$ if Equations (1) and (10) are satisfied. Thus, using this presentation of experimental data, one may check the linearity of the curve, determine the slope $S_{\mathrm{lp}}$ of its linear portion, and estimate the scattering exponent

(14) $$s=-S_{\mathrm{lp}}-1.$$

For differentiation of the experimental curve of measured optical density $D_{m}\left(\lambda \right)$, any graphical editor can be used.

To demonstrate the applicability of this new method, we apply it to several different NGs with crystalline phases of different compositions, structures, sizes, and volume fractions and compare values of the scattering exponent with those obtained using one of the three methods described in Section 1. The implementation of the new method requires numerical data on the measured optical density, which cannot be extracted with a sufficient degree of accuracy from the graphic material presented in the articles, i.e., we are forced to use only our own spectra of the measured optical density. Five NGs were studied in our group earlier by one of the three methods and the obtained values of the scattering exponent were presented in publications. This list was supplemented with two new NGs studied in this work by one of the traditional methods and one of the new method (see Section 2.2.4 and Section 2.4.2).

In all the cases, absorption spectra were measured on a Shimadzu UV-3600 spectrophotometer in the spectral range from 190 to 3300 nm. The wavelength step of measurement, ∆λ, will be indicated separately in each case.

### 2.2. Application of the Novel Method and Comparison to the Results with Those Obtained Using the First Method

#### 2.2.1. Phase-Separated Sodium Borosilicate Glass

In Ref. [26], the extinction coefficient of the phase-separated sodium borosilicate glass denoted G2 was studied by the first method using measured spectra of optical density for two samples of different thicknesses and Equation (5).

The initial glass of the composition 13.9Na_2_O·36.0B_2_O_3_·50.1SiO_2_ (mol% by analysis) with the weight of 500 kg was melted in an industrial furnace at a temperature of 1250 °C, cooled in the crucible from 1250 to 500 °C for 70 h, and held at that temperature for 8 h, after which it was allowed to cool to room temperature at a rate of 4 °C per h. The phase-separated glass G2 was prepared from the initial glass by heat treatment for 10 h at 610 °C. The wavelength step of absorption spectrum recording was ∆λ=2 nm. It was found that for λ>360 nm, the spectral dependence of the extinction coefficient is described by Equation (1) with the value of the scattering exponent:

(15) s=6.9±0.1

(see Equation (4) and Figure 2a of Ref. [26]). The deviation from this behavior at λ<360 nm was related to the light absorption.

**Figure 1:**
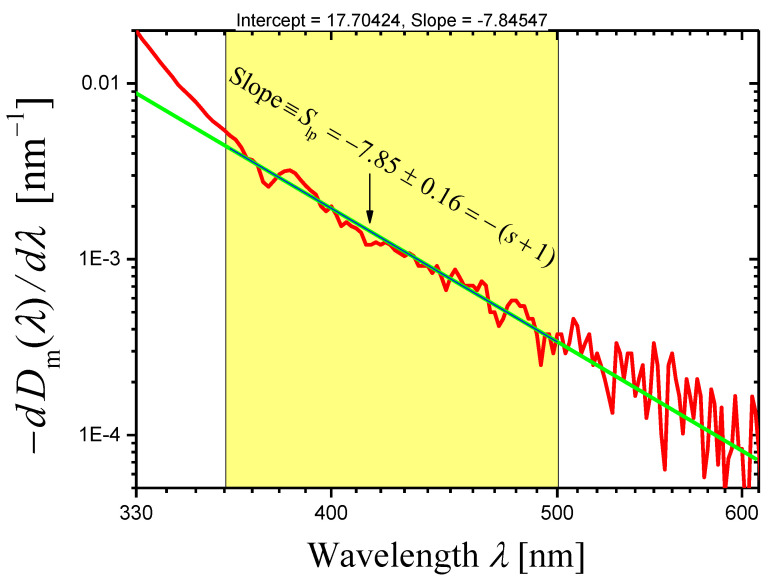
A log–log plot of the dependence of $\left(-dD_{m}(\lambda )/d\lambda \right)$ on λ constructed for the measured optical density $D_{m}(\lambda )$ of the glass G2 sample with the thickness $h_{2}=10.00\;\mathrm{mm}$ (red curve). The green straight line presents the linear least squares approximation of the curve in the spectral range of 360−500 nm.

Figure 1 presents a log–log plot of the dependence of $\left(-dD_{m}(\lambda )/d\lambda \right)$ on λ where $D_{m}(\lambda )$ is the measured optical density for the thick sample of the glass G2 (thickness $h_{2}=10.00\;\mathrm{mm}$; $D_{m}(\lambda )$ is shown as curve *2* in Figure 1b of Ref. [26]). Absorption is observed at wavelengths λ<360 nm [26]. At λ>500 nm, fluctuations of the derivative $\left(-dD_{m}(\lambda )/d\lambda \right)$ are very strong, and for some wavelengths, its values are even negative. In the spectral range of 360−500 nm, the curve in Figure 1 can be approximated by a straight line, and its linear least squares approximation is also shown in Figure 1. The slope of the straight line is $S_{\mathrm{lp}}=-7.85\pm 0.16$, so the novel method gives the value of the scattering exponent (Equation (14))

(16) s=6.85±0.16,

which coincides well with the value obtained by the first method (Equation (15)). It should be noted that the error of $S_{\mathrm{lp}}$ and s is taken to be twice the standard error, which is determined in the course of the linear least squares approximation.

#### 2.2.2. Gahnite-Based Zinc Aluminosilicate Glass-Ceramic Studied in Reference [34] and Denoted as GC1

The initial glass of the composition 25ZnO·25Al_2_O_3_·50SiO_2_ (mol% by synthesis) doped by a mixture of 5TiO_2_ and 5ZrO_2_ as nucleating agents [37] was subjected to two-step heat treatment (750 °C, 6 h + 1000 °C, 6 h). As a result, a glass-ceramic was formed consisting of ZnAl_2_O_4_, ZrO_2_, and ZrTiO_4_ nanocrystals with diameters *d* = 14, 12 and 24 nm, respectively, distributed in amorphous matrix whose composition is close to that of silica glass [38].

The spectra of the optical density of the samples of this glass-ceramic denoted as GC1 were measured with the wavelength step ∆λ=2 nm and studied in Ref. [34]. The optical densities $D_{im}\left(\lambda \right)$ for three samples with thicknesses $h_{i}$ ($h_{1}=0.27\;\mathrm{mm}$, $h_{2}=0.77\;\mathrm{mm}$, and $h_{3}=3.02\;\mathrm{mm}$) are shown in Figure 4 of Ref. [34]. It was noted that there is no absorption at λ>380 nm and the attenuation of light in the wavelength range 380–1000 nm is caused by scattering. For three pairs of samples ($(i,i^{\prime })=$ (1,2), (1,3), and (2,3)), the extinction coefficients $\alpha_{{ii}^{\prime }}(\lambda )$ were determined by the first method using the measured spectra of optical densities and Equation (5) (see Figure 5 in Ref. [34]). The obtained dependences $\alpha_{{ii}^{\prime }}(\lambda )$ can be described by Equation (1) in certain spectral ranges. The values of the scattering exponent were found to be:

(17) $$s≅4.6{(\alpha }_{1,2}),\;s≅4.5{(\alpha }_{1,3},\;\alpha_{2,3})$$

(see Equation (21) in Ref. [34]).

**Figure 2:**
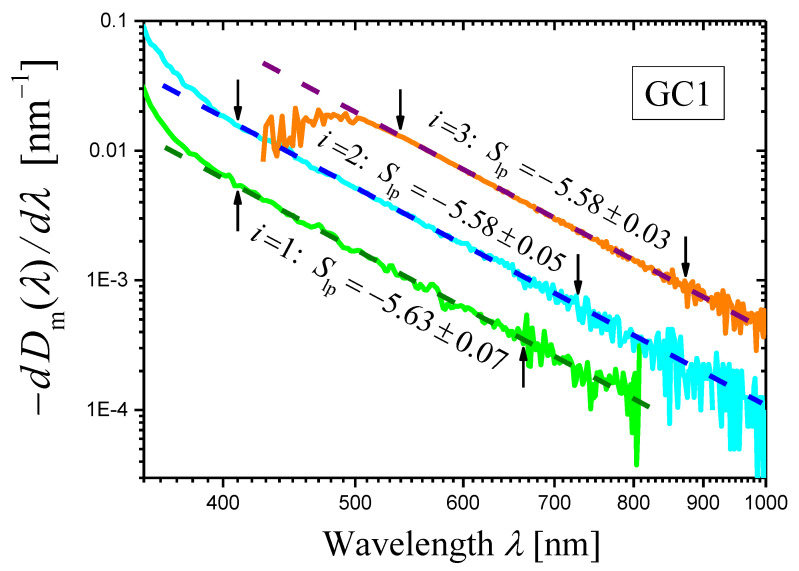
Log–log plots of the dependences of $\left(-dD_{im}(\lambda )/d\lambda \right)$ on λ (curves) constructed for the measured optical densities $D_{im}(\lambda )(i=1,2,3)$ of three samples of the glass-ceramic GC1. The thicknesses of samples are: $h_{1}=0.27\;\mathrm{mm}$ (green curve), $h_{2}=0.77\;\mathrm{mm}$ (cyan curve), and $h_{3}=3.02\;\mathrm{mm}$ (orange curve). The portion of each curve limited by arrows is approximated by a dashed straight line using the least squares method, and the slope $S_{\mathrm{lp}}$ of the straight line is indicated in the figure.

The derivatives of the measured optical densities $D_{im}\left(\lambda \right)(i=1,2,3)$ as functions of wavelength λ are shown in Figure 2 as log–log plots. One can see that certain portions of the curves may be presented as straight lines. In the ranges limited by arrows in Figure 2, each curve was approximated by a straight line using the least squares method. The results of the approximation are presented by dashed straight lines, and the slopes of the lines are indicated. Using these slopes $S_{\mathrm{lp}}$ and Equation (14), we can write the scattering exponent values obtained by the proposed novel method as:

(18) $$s=4.63\pm 0.07\left(i=1\right),\;s=4.58\pm 0.05\left(i=2\right),\;s=4.58\pm 0.03\left(i=3\right),$$

which coincide well with the values obtained by the first method (Equation (17)).

#### 2.2.3. Gahnite-Based Zinc Aluminosilicate Glass-Ceramic Studied in Ref. [34] and Denoted as GC3

To prepare the glass-ceramic denoted as GC3 in Ref. [34], the initial glass of the same composition as that for the GC1 one (Section 2.2.2) and melted in the same conditions was subjected to two-step heat treatment (800 °C, 6 h + 1050 °C, 6 h), with the temperature of both steps being 50 °C higher than for GC1. The phase composition of the GC3 was the same as for GC1, and the sizes of the crystals were somewhat larger than those in GC1 (the difference was less than 50%) [34].

The optical density $D_{im}\left(\lambda \right)$ was measured with the wavelength step ∆λ=2 nm for two GC3 samples of thickness $h_{1}=2.11\;\mathrm{mm}$ and $h_{2}=3.02\;\mathrm{mm}$ (Figure 7 of Ref. [34]). For this pair of samples, the extinction coefficient $\alpha_{1,2}(\lambda )$ was determined by the first method using the measured spectra of optical densities and Equation (5) (see Figure 8 in Ref. [34]). The obtained dependence $\alpha_{1,2}(\lambda )$ can be described by Equation (1) in the spectral range 440–870 nm, and the following value of the scattering exponent was found:

(19) $$s≅5.3\;{(\alpha }_{1,2}).$$

(see Equation (27) in Ref. [34]).

**Figure 3:**
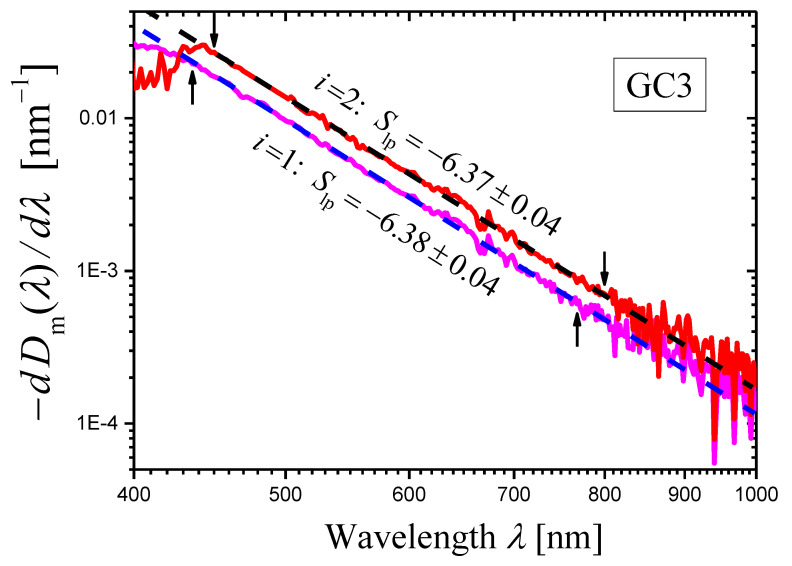
Log–log plots of the dependences of $\left(-dD_{im}(\lambda )/d\lambda \right)$ on λ (curves) constructed for the measured optical densities $D_{im}(\lambda )(i=1,2)$ of two samples of the glass-ceramic GC3. The thicknesses of the samples are: $h_{1}=2.11\;\mathrm{mm}$ (magenta curve) and $h_{2}=3.02\;\mathrm{mm}$ (red curve). The portion of each curve limited by arrows is approximated by a dashed straight line using the least squares method, and the slope $S_{\mathrm{lp}}$ of the straight line is indicated in the figure.

The derivatives of the measured optical densities $D_{im}\left(\lambda \right)(i=1,2)$ as functions of wavelength λ are shown in Figure 3 as log–log plots. One can see that certain portions of the curves can be presented as straight lines. In the ranges limited by arrows in Figure 3, each curve was approximated by a straight line using the least squares method. The results of the approximation are presented by dashed straight lines, and the slopes of the lines are indicated. Using these slopes $S_{\mathrm{lp}}$ and Equation (14), we can write the scattering exponent values obtained by the novel method as:

(20) $$s=5.38\pm 0.04\left(i=1\right),\;s=5.37\pm 0.04\left(i=2\right),$$

which coincides well with the value obtained by the first method (Equation (19)).

#### 2.2.4. Spinel-Based Magnesium Aluminosilicate Glass-Ceramic

In Refs. [39,40], a number of glass-ceramics were developed on the basis of glass with the composition 20MgO·20Al_2_O_3_·60SiO_2_ (mol% by synthesis) doped by 10TiO_2_ as a nucleating agent (MAS composition). Initial glass 300 g in weight was melted in a laboratory electric furnace with SiC heating elements in crucibles made of quartz ceramics at 1560 °C for 8 h with stirring, quenched by pouring onto a cold metal plate and annealed at 660 °C. The glass-ceramic was prepared from the initial MAS glass by two-step heat treatment (750 °C, 6 h + 1000 °C, 6 h). It will be denoted as MAS-GC. This glass-ceramic contained crystals of magnesium aluminate spinel with the average size of ≈8 nm and of magnesium aluminotitanate solid solution with the average size of ≈15 nm distributed in the highly siliceous residual glass.

Here, we study the scattering exponent of this glass-ceramic by using the first and new methods.

**Figure 4:**
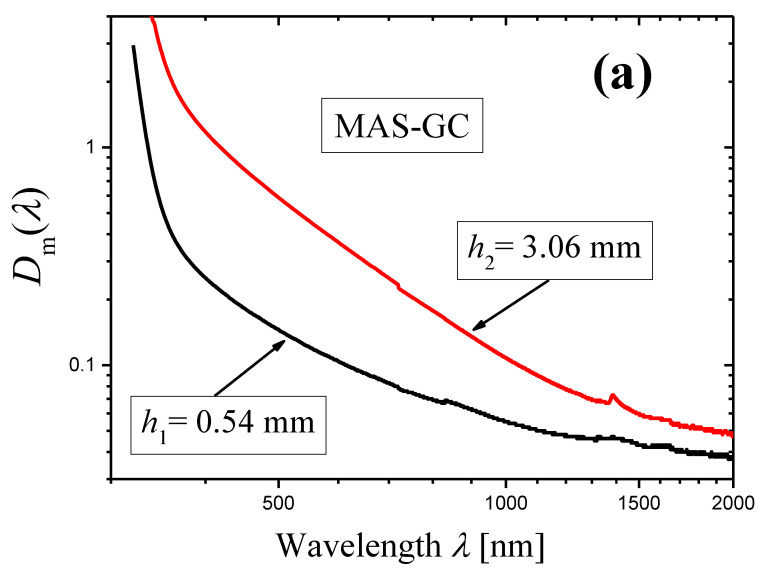

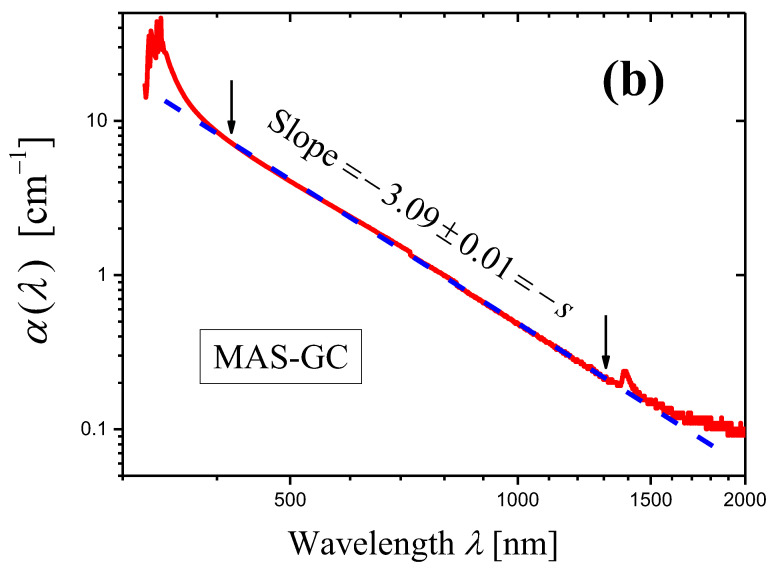
(**a**): Log–log plots of the measured optical densities for two samples of the MAS-GC with thicknesses indicated in the figure. (**b**): A log–log plot of wavelength dependence of the extinction coefficient α(λ) determined by Equation (5) on the basis of the data presented in Figure 4a (the first method).

The optical density $D_{im}\left(\lambda \right)$ was measured with the wavelength step ∆λ=1 nm for two samples of the MAS-GC of thickness $h_{1}=0.54\;\mathrm{mm}$ and $h_{2}=3.06\;\mathrm{mm}$ (Figure 4a). The results of these measurements were used for calculation of extinction coefficient α(λ) by Equation (5) (the first method). The dependence α(λ) is shown in Figure 4b as a log–log plot.

In the wavelength range ≈ 400–1350 nm, the curve in Figure 4b demonstrates a linear portion ascribed to light scattering. Absorption appears at λ≲400 nm. The slope of the linear portion was determined by the least squares method in the spectral range shown by arrows and is equal to −3.09 ± 0.01. Thus, the first method demonstrates that the scattering coefficient is described by Equation (1) with the scattering exponent:

(21) s=3.09±0.01.

**Figure 5:**
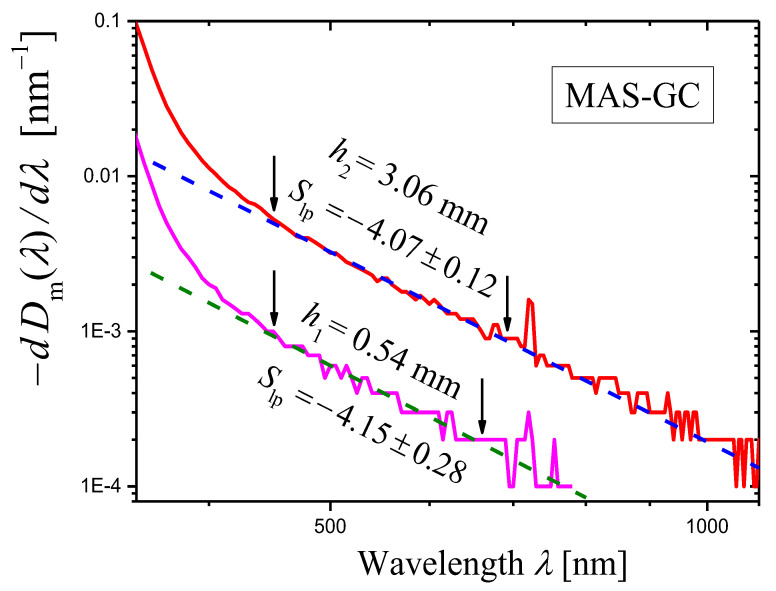
Log–log plots of the dependences of $\left(-dD_{im}(\lambda )/d\lambda \right)$ on λ (curves) constructed for the measured optical densities $D_{im}(\lambda )(i=1,2)$ of two samples of the MAS glass-ceramic studied in the present work. The thicknesses of the samples are: $h_{1}=0.54\;\mathrm{mm}$ (magenta curve) and $h_{2}=3.06\;\mathrm{mm}$ (red curve). The portion of each curve limited by arrows is approximated by a straight dashed line using the least squares method, and the slope $S_{\mathrm{lp}}$ of the straight line is indicated in the figure.

It should be noted that the initial spectra of the measured optical densities $D_{im}\left(\lambda \right)(i=1,2)$ presented in Figure 4a were recorded with the step Δλ=1 nm. They were used for the construction of Figure 4b. However, the differentiation of these spectra led to strong oscillations. To reduce oscillations of the derivatives, we recalculated the initial spectra to a step Δλ=5 nm (also see Section 3.2). The derivatives of the recalculated spectra are shown in Figure 5 as functions of wavelength in a log–log scale. One can see that certain portions of the curves may be presented as straight lines. In the ranges limited by arrows in Figure 5, each curve was approximated by a straight line using the least squares method. The results of the approximation are presented by dashed straight lines, and the slopes of the lines are indicated. Using these slopes $S_{\mathrm{lp}}$ and Equation (14), we can write the scattering exponent values obtained by the novel method as:

(22) $$s=3.15\pm 0.28\left(i=1\right),\;s=3.05\pm 0.12\left(i=2\right),$$

which coincides well with the value obtained by the first method (Equation (21)).

### 2.3. Application of the Novel Method and Comparison of the Results with Those Obtained in Ref. [35] for Glass-Ceramics Based on Sr^II^Nb^IV^O_3_ Crystals Using the Second Method

Let us consider glass-ceramics GC20 and GC40 prepared and characterized by U. Gangadharini et al. in Ref. [41]. The light scattering in these glass-ceramics was studied in Ref. [35]. The initial glass of the composition 53.75SiO_2_—18.25K_2_O—9Bi_2_O_3_—9SrO—9Nb_2_O_5_—0.5CeO_2_—0.5Eu_2_O_3_ (mol%) was heat-treated at 450 °C for 10 h and then at 500 °C for 20 and 40 h to prepare the glass-ceramics GC20 and GC40, respectively [41].

The glass-ceramics contain Sr^II^Nb^IV^O_3_ crystals (JCPDS file card No. 79-0625) with sizes of 20–25 nm [41] and are characterized by strong light scattering in visible and IR ranges.

For each glass-ceramic, refractive indices n were measured at four wavelengths of 532, 632.8, 1064, and 1552 nm [41]. In Ref. [35], these data were approximated by an analytical function for each glass-ceramic to obtain the refractive index n(λ) depending on the wavelength. Using the measured optical density $D_{m}(\lambda )$ (∆λ=1 nm) of the sample with thickness h=2.00 mm, the refractive index n(λ), and Equations (6) and (4), the internal optical density D(λ) of the sample and the extinction coefficient α(λ) of a given glass-ceramic were evaluated [35]. The conclusion was made that the wavelength dependence α(λ) for each glass-ceramic satisfies Equation (1) in a wide spectral range, and the following values of the scattering coefficient were found:

(23) $$s=5.1\pm 0.1\left(\mathrm{GC}20\right),\;s=5.4\pm 0.1\left(\mathrm{GC}40\right).$$

**Figure 6:**
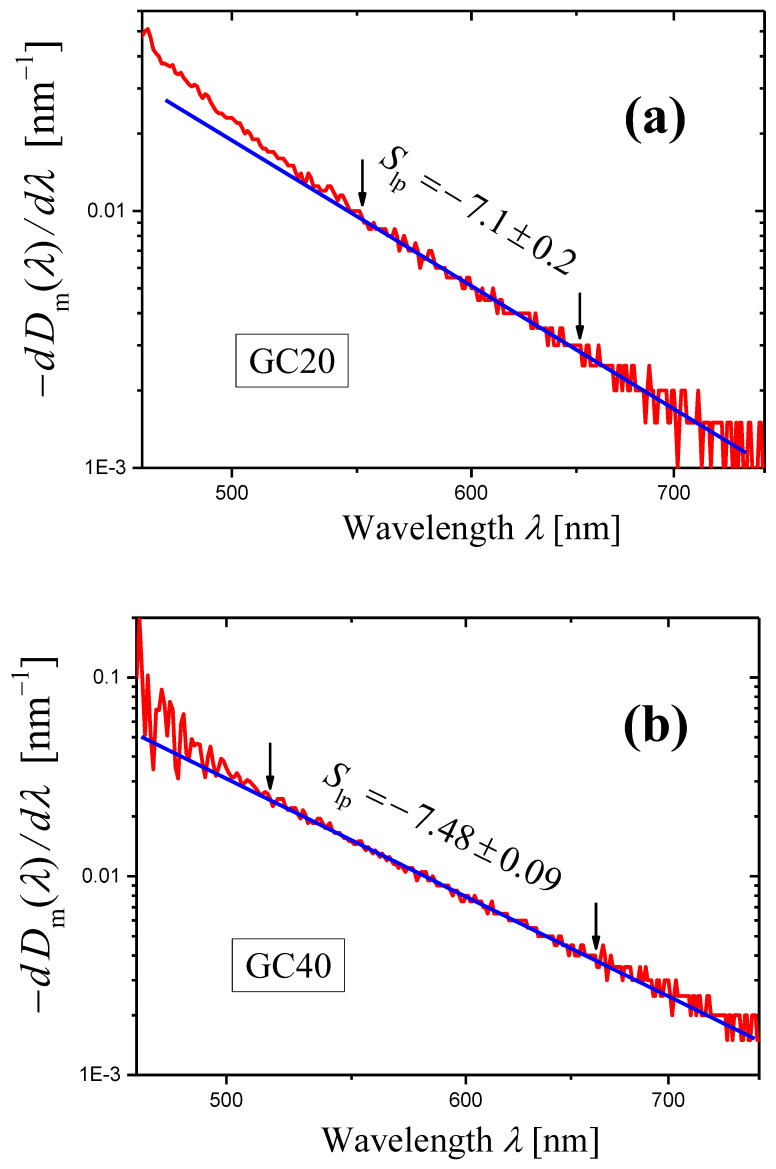
Log–log plots of the dependences of $\left(-dD_{im}(\lambda )/d\lambda \right)$ on λ (red curves) constructed for the measured optical densities $D_{m}(\lambda )$ of the samples of the glass-ceramics GC20 (**a**) and GC40 (**b**). The samples have the same thickness h=2.00 mm. The portion of each curve limited by arrows is approximated by blue straight line using the least squares method, and the slope $S_{\mathrm{lp}}$ of the straight line is indicated in the figure.

For the glass-ceramics GC20 and GC40, the derivatives of measured optical densities $D_{m}\left(\lambda \right)$ as functions of wavelength λ are shown in Figure 6 in the form of log–log plots. Certain portions of the curves may be presented as straight lines. In the ranges limited by arrows in Figure 6, each curve was approximated by a straight line using the least squares method. The results of the approximation are presented by straight lines, and the slopes of the lines are indicated. Using these slopes $S_{\mathrm{lp}}$ and Equation (14), we can write the scattering exponent values obtained by the novel method as:

(24) $$s=6.1\pm 0.2\left(\mathrm{GC}20\right),\;s=6.48\pm 0.09\left(\mathrm{GC}40\right).$$

In this case, the results obtained by the new method differ significantly from those determined by the second traditional method (compare Equations (23) and (24)).

### 2.4. Application of the Novel Method and Comparison of the Results with Those Obtained Using the Third Method

#### 2.4.1. Er-Doped Potassium Zinc Aluminosilicate Glass-Ceramic Containing ZnO Nanocrystals

The structure and optical properties of glass-ceramics containing ZnO nanocrystals and prepared by crystallization of the initial glass with composition 14K_2_O—32ZnO—14Al_2_O_3_—40SiO_2_ + 1.5Er_2_O_3_ (mol%) were studied in Ref. [36].

The glass of 400 g in weight was melted in quartz ceramic crucible in a laboratory electric furnace at 1590 °C for 6 h with stirring, cast onto a cold metal plate and annealed at 500 °C. To obtain glass-ceramics, the initial glass was subjected to different heat treatments. The optical density spectra of the samples of the initial glass and glass-ceramics were measured with the wavelength step ∆λ=2 nm. Here, we consider glass-ceramic denoted as ZnOEr-GC, which was obtained by the successive heat treatment (2 + 1) h at 750 °C. This glass-ceramic contains the ZnO nanocrystals with the average size of 30 ± 2 nm [36]. Among the glass-ceramics studied in Ref. [36], the ZnOEr-GC one demonstrates the maximum light scattering.

Using the third method, the following value of the scattering exponent was obtained for ZnOEr-GC (see Table 1 in Ref. [36]):

(25) $$s=7.0\pm 0.2\left(\mathrm{ZnOEr-GC}\right).$$

**Figure 7:**
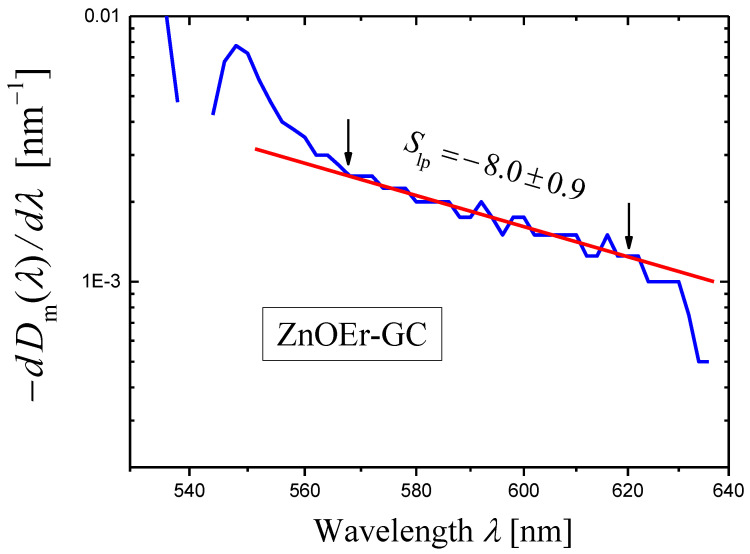
A log–log plot of the dependence of $\left(-dD_{im}(\lambda )/d\lambda \right)$ on λ (blue curve) constructed for the measured optical densities $D_{m}(\lambda )$ of sample of the glass-ceramic ZnOEr-GC. The sample thickness h=1.06 mm. The portion of the curve limited by arrows is approximated by a red straight line using the least squares method, and the slope $S_{\mathrm{lp}}$ of the straight line is indicated in the figure.

The application of the novel method is complicated by the presence of numerous absorption peaks of erbium ions in the spectral range where light scattering is significant. The widest spectral range free from absorption peaks is the range of 568–620 nm. In this range the log–log plot of the dependence of $\left(-dD_{m}(\lambda )/d\lambda \right)$ on λ was approximated by a straight line using the least squares method (Figure 7). Since this spectral range is narrow, the error in determining the slope $S_{\mathrm{lp}}$ and the scattering exponent s is significant:

(26) $$s=7.0\pm 0.9\left(\mathrm{ZnOEr-GC},\;\mathrm{the}\;\mathrm{novel}\;\mathrm{method}\right).$$

This value of s coincides well with the value obtained by the third method (Equation (25)).

#### 2.4.2. Yb-Doped Potassium Zinc Aluminosilicate Glass-Ceramic Containing ZnO Nanocrystals

We synthesized the initial glass with the composition 14K_2_O·32ZnO·14Al_2_O_3_·40SiO_2_ +1.5Yb_2_O_3_ (mol%) and prepared the glass-ceramic (ZnOYb-GC) by heat treatment at 750 °C for 2 h [42]. This ZnOYb-GC contains ZnO nanosized crystals.

**Figure 8:**
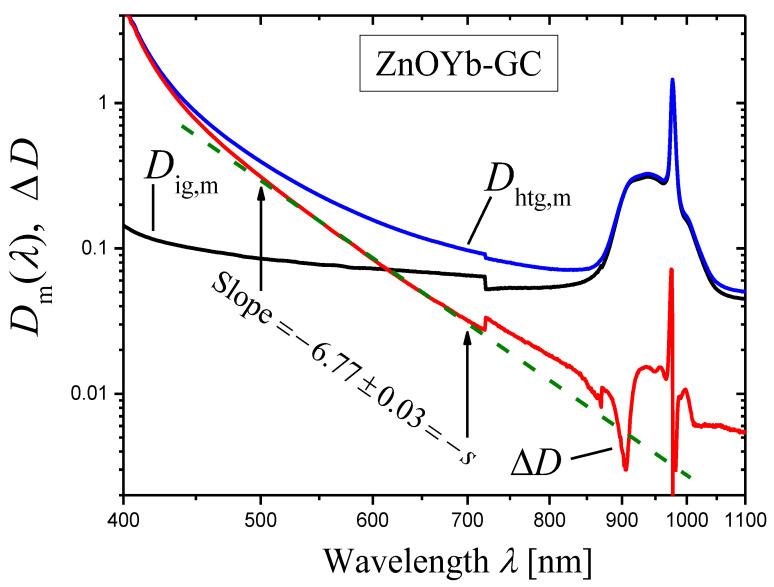
The measured optical densities $D_{\mathrm{ig},m}(\lambda )$ of the sample of the initial glass (with thickness h=3.00 mm) (black curve) and $D_{\mathrm{htg},m}\left(\lambda \right)$ of the sample of the ZnOYb-GC (with thickness h=3.02 mm) (blue curve), see Equation (7), and their difference $∆D\left(\lambda \right)$ (red curve) (Equation (8)) (log–log plot). The slope of the linear portion of $∆D\left(\lambda \right)$ curve was determined by the least squares method in the spectral range shown by arrows, and is indicated in the figure.

Optical densities $D_{\mathrm{ig},m}(\lambda )$ of the sample of the initial glass (with thickness h=3.00 mm) and $D_{\mathrm{htg},m}\left(\lambda \right)$ of the sample of the ZnOYb-GC (with thickness h=3.02 mm), see Equation (7), were measured with the wavelength step ∆λ=1 nm. Then, their difference $∆D\left(\lambda \right)$ (Equation (8)), which is proportional to the extinction coefficient (Equation (9)), was calculated (Figure 8, the log–log plot). In the wavelength range ≈ 500–850 nm, the curve of $∆D\left(\lambda \right)$ in Figure 8 demonstrates a linear portion ascribed to light scattering. The slope of this linear portion was determined by the least squares method in the spectral range shown by arrows and was found to be equal to −6.77 ± 0.03. Thus, one can state that the scattering coefficient is described by Equation (1) with the scattering exponent value

(27) s=6.77±0.03.

**Figure 9:**
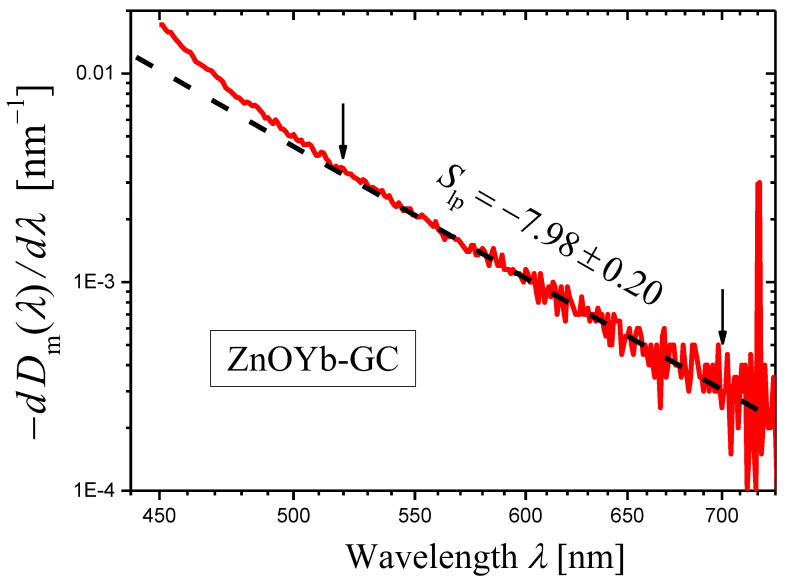
A log–log plot of the dependence of $\left(-dD_{im}(\lambda )/d\lambda \right)$ on λ (red curve) constructed for the measured optical density $D_{m}(\lambda )$ of sample of the glass-ceramic ZnOYb-GC with the thickness h=3.02 mm. The portion of the curve limited by arrows is approximated by a black dashed straight line using the least squares method, and the slope $S_{\mathrm{lp}}$ of the straight line is indicated in the figure.

The wavelength dependence of the derivative of the measured optical density $D_{\mathrm{htg},m}\left(\lambda \right)$ for the ZnOYb-GC sample is shown in Figure 9 as the log–log plot. A certain portion of the curve may be presented as a straight line. In the ranges limited by arrows in Figure 9, the curve was approximated by a straight line using the least squares method. The result of the approximation is presented by a dashed straight line, and the slope of the line is indicated. Using this slope $S_{\mathrm{lp}}$ and Equation (14), we can write the scattering exponent value obtained by the novel method as:

(28) s=6.98±0.20

which coincides well with the value obtained by the third method (Equation (27)).

## 3. Discussion

In this part of the work, we discuss the features of the application of the proposed new, simple method for the estimation of the scattering exponent of NGs.

### 3.1. Comparison of the Scattering Exponent Values Obtained by the New and Traditional Methods

The scattering exponent values obtained by the new and three different previously developed methods were compared. In most of the examples given in Section 2, the novel method gives good estimates for the scattering exponent. This conclusion was made based on a comparison of the scattering exponent for NGs with amorphous and crystalline inhomogeneities of different nature, different sizes, and with different volume fractions of inhomogeneity regions.

A significant difference between the values of the scattering exponent obtained by the novel and the traditional methods is found only in the examples given in Section 2.3 for glass-ceramics based on Sr^II^Nb^IV^O_3_ crystals (compare Equations (23) and (24)). Let us try to find the reasons for this discrepancy. In Section 2.3, we applied the novel method in the wavelength ranges from 552 to 652 nm (GC20, Figure 6a) and from 517 to 663 nm (GC40, Figure 6b). Since the ranges are narrow and there are strong oscillations of the curves, we could not find non-linear behavior of the curves in these ranges. However, Figure 4c,d in Ref. [35] show that Equation (1) is not satisfied in these ranges, and thus, portions of straight lines cannot be found on curves in Figure 6a,b. We will illustrate this conclusion below.

**Figure 10:**
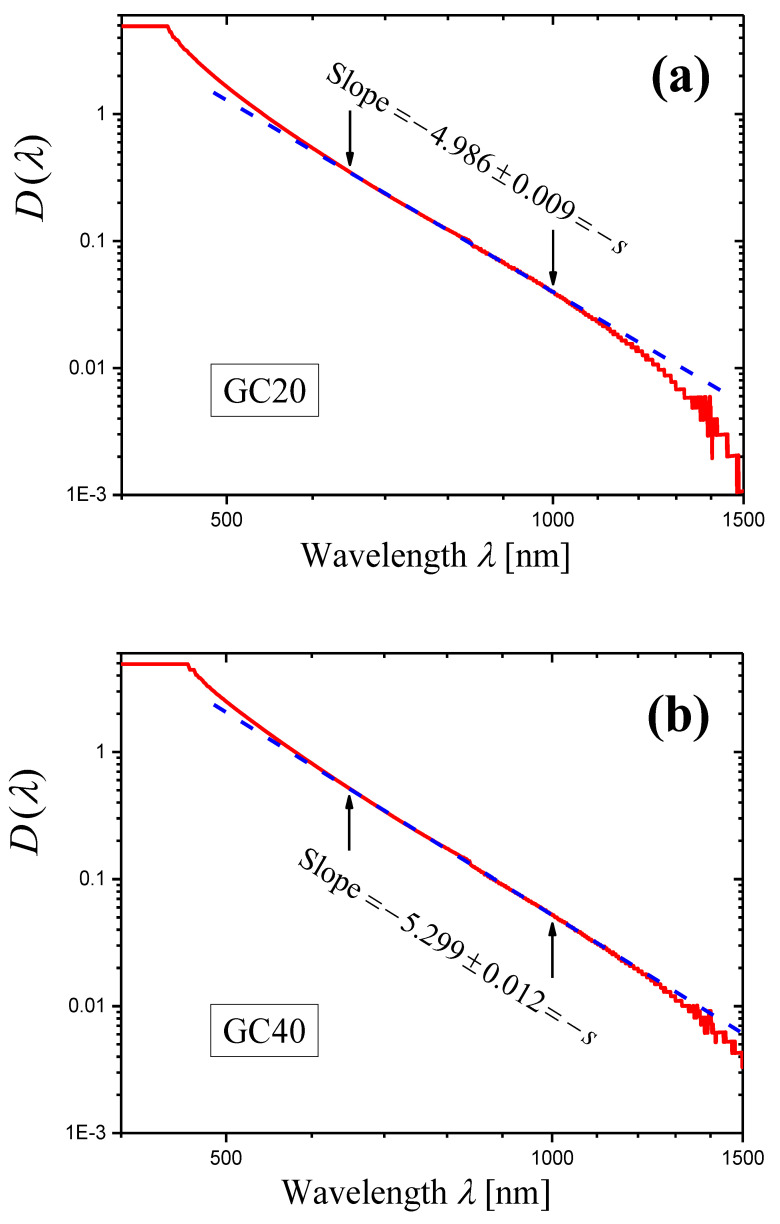
Log–log plots of the internal optical density D(λ) (red curves) for samples of the glass-ceramics GC20 (**a**) and GC40 (**b**). The samples have the same thickness h=2.00 mm. The portion of each curve limited by arrows is approximated by red dashed straight line using the least squares method, and the slope of the straight line is indicated in the figure.

To compare the novel and the second method for the glass-ceramics GC20 and GC40, we should first refine the results obtained by the second method in Ref. [35]. Figure 10a,b shows log–log plots of the internal optical densities D(λ) for 2 mm-thick samples of GC20 and GC40, respectively. The internal optical density was calculated by Equation (6) using the measured optical density $D_{m}\left(\lambda \right)$ and analytical expressions for the refractive indices $n\left(\lambda \right)$ [35]. Both curves presented in Figure 10 demonstrate good linearity in the range from 650 to 1000 nm, and this range will be used here and below for application of the least squares method and for determination of slopes of the linear portion of curves. For this range, we obtain the more accurate values of the scattering exponent:

(29) $$s=4.986\pm 0.009\left(\mathrm{GC}20\right),\;s=5.299\pm 0.012\left(\mathrm{GC}40\right),$$

than those given in Ref. [35] (Figure 4a,b) and presented above (see Equation (23)).

Because of strong oscillations, the derivatives calculated with the wavelength step Δλ=1 nm and shown in Figure 6 become meaningless in the spectral range from 650 to 1000 nm where Equation (1) is satisfied (Figure 10) and the novel method will be used. It should be noted that oscillations are related to measurement errors. To obtain a more or less regular derivative curve in the vicinity of wavelength λ, the difference ${\Delta D_{m}\left(\lambda ,\Delta \lambda \right)=D}_{m}\left(\lambda \right)-D_{m}\left(\lambda +\Delta \lambda \right)$ must be substantially greater than the measurement error, $\delta D_{m}$. Otherwise, the derivative will strongly oscillate. To reduce oscillations, we increased the wavelength step, i.e., $\Delta D_{m}\left(\lambda ,\Delta \lambda \right)$. The following values of the step were used:

(30) Δλ=2,5,10,20,30,50 nm.

It should be noted that in all the cases, we used the same initial spectrum of the measured optical density recorded with the step Δλ=1 nm and recalculated it to the spectrum with a given step by a simple FORTRAN program.

If Δλ≥5 nm, the derivative curves for both glass-ceramics GC20 and GC40 are acceptable for analysis in the spectral range of 650–1000 nm. For the presentation, we used the step Δλ=20 nm, for which the errors in slopes $S_{\mathrm{lp}}$ are minimal. It should be noted that the spread of slope values calculated with different steps does not exceed 0.18 (GC20) and 0.07 (GC40) and is less than the minimal slope error 0.24 determined for both glass-ceramics at the step Δλ=20 nm.

**Figure 11:**
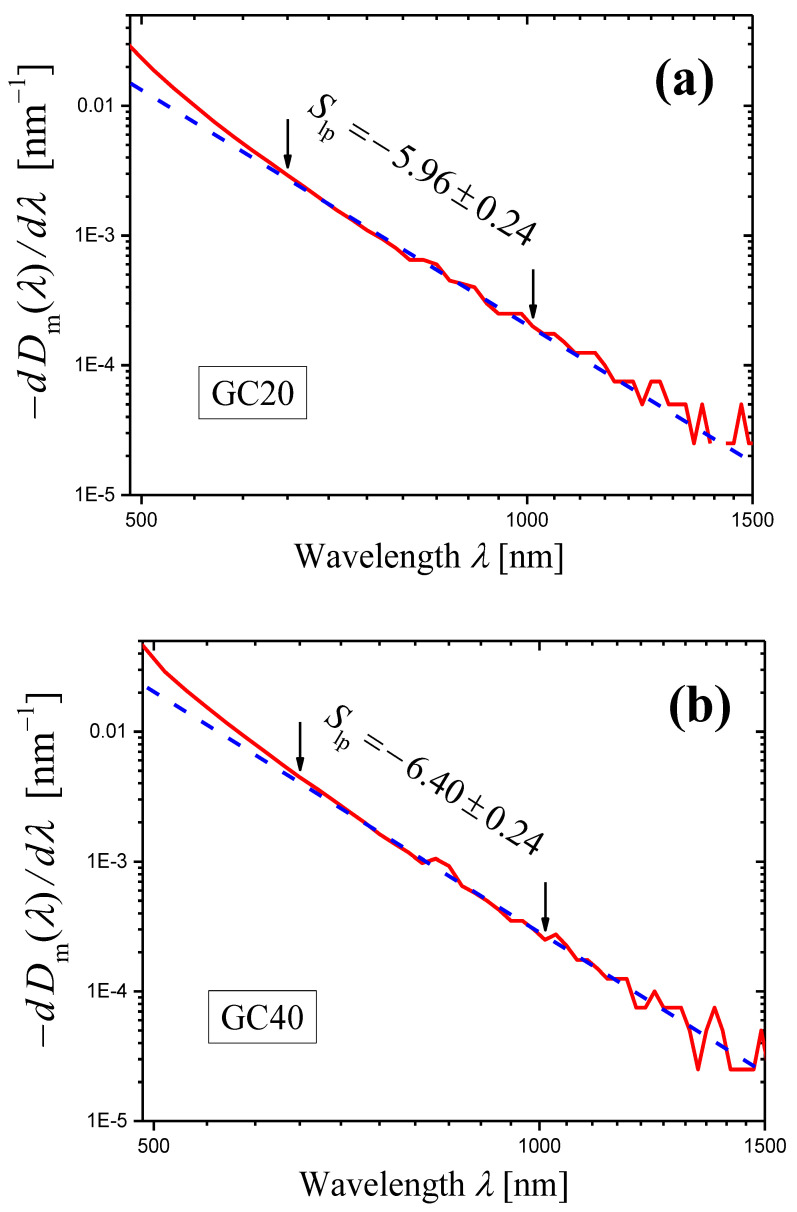
Log–log plots of the dependences of $\left(-dD_{im}(\lambda )/d\lambda \right)$ on λ (red curves) constructed for the measured optical densities $D_{m}(\lambda )$ of samples of the glass-ceramics GC20 (**a**) and GC40 (**b**). Notice that the spectral range is wider than in Figure 6 for the same glass-ceramics; the wavelength step Δλ=20 nm is greater than the step Δλ=1 nm used to draw the curves in Figure 6.

The log–log plots of the dependence of $\left(-dD_{m}(\lambda )/d\lambda \right)$ on λ obtained with the step Δλ=20 nm for the glass-ceramics GC20 and GC40 are shown in Figure 11. One can see that the curves are non-linear at wavelengths λ≈600 nm, so the analysis carried out in Section 2.3 should have led to erroneous results. The curves were approximated by straight lines in the range of 650–1000 nm by the least squares method. Using the obtained slopes $S_{\mathrm{lp}}$ and Equation (14), we can write the scattering exponent values obtained by the novel method as:

(31) $$s=4.96\pm 0.24\left(\mathrm{GC}20\right),\;s=5.40\pm 0.24\left(\mathrm{GC}40\right).$$

These values are in good agreement with the values given in Equation (29).

### 3.2. On the Choice of the Wavelength Step in the Spectrum of the Measured Optical Density

Basing on the results presented in Section 3.1, we can conclude that the novel method leads to correct values of the scattering exponent for the glass-ceramics GC20 and GC40 if it is applied in the proper wavelength range, and the proper wavelength step is chosen.

The problem of the choice of the wavelength step was also discussed in Section 2.2.4 for the MAS-GC.

Thus, the choice of the proper step Δλ is an important element in the implementation of the method.

**Figure 12:**
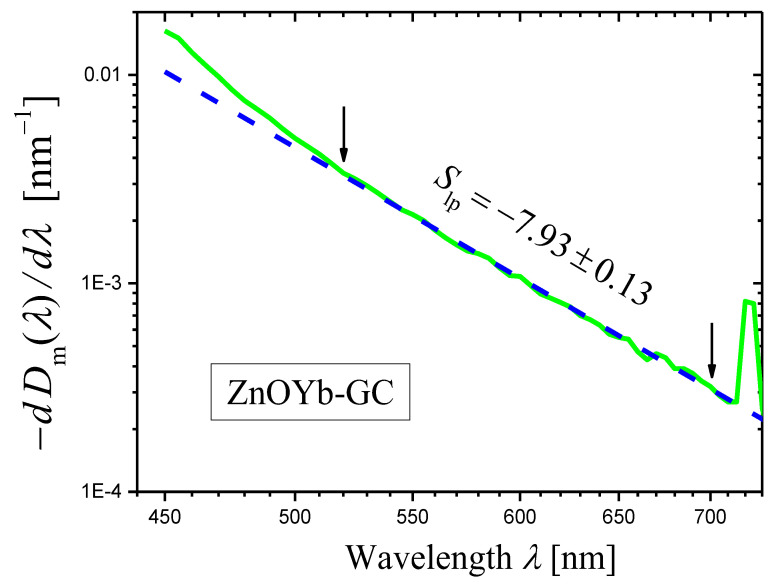
A log–log plot of the dependence of $\left(-dD_{im}(\lambda )/d\lambda \right)$ on λ (green curve) constructed for the measured optical density $D_{m}(\lambda )$ of sample of the glass-ceramic ZnOYb-GC with the thickness h=3.02 mm. The portion of the curve limited by arrows is approximated by blue dashed straight line using the least squares method, and the slope $S_{\mathrm{lp}}$ of the straight line is indicated in the figure. The wavelength step Δλ=5 nm was used.

To illustrate the effect of the step size on the $\left[\log_{10}(-dD_{m}(\lambda )/d\lambda )]-[\log_{10}\left(\lambda \right)\right]$ curve and the slope of its linear portion, we take the example of ZnOYb-GC (Section 2.4.2) for which the step Δλ=1 nm was used in Figure 9. For the construction of Figure 12, we used the step Δλ=5 nm. It can be seen that the curve in Figure 12 is much smoother than the curve shown in Figure 9. However, the value of the scattering exponent determined from Figure 12, s=6.93±0.13 agrees well with the value s=6.98±0.20 (Equation (28)) determined from Figure 9 for the same wavelength range of 520–700 nm.

If we want to find the most accurate value for the scattering exponent, we can plot the derivative curves for several step values in a log–log scale, fit a portion of each curve with a straight line using the least squares method, and choose the step value corresponding to the smallest standard deviation of the slope $S_{\mathrm{lp}}$. As noted above, such a procedure was implemented for the glass-ceramics GC20 and GC40.

### 3.3. On the Choice of the Sample Thickness

A comparison of the curves in Figure 5 and qualitative considerations show that the oscillations decrease as the sample thickness increases. Accordingly, the error in determining the slope of the curve decreases with increasing the sample thickness (also see Figure 2). However, it should be kept in mind that if a sample is too thick, the condition specified by Equation (3) may be violated for the spectral range of interest. This violation manifests itself in a slowdown in the increase in the measured optical density with decreasing wavelength [34]. The consequence of this slowdown can be seen for sample 3 in Figure 2 at λ<503 nm, and for both samples in Figure 3 at λ≲440 nm. Fortunately, in these cases the effect takes place at the edge of the linear portion of the curves.

### 3.4. Applicability of the New Method to the Cases of Wavelength-Dependent Reflection Losses

When formulating the new method, we assumed that the optical density of reflection losses is independent of wavelength (Equation (5)). In most cases, this assumption is not met. Therefore, it is important to estimate how the wavelength dependence of the optical density of reflection losses affects the results of applying the new method.

If the optical density of reflection losses is a function of wavelength, $D_{\mathrm{refl}}\left(\lambda \right)$, Equations (12) and (13) should be replaced by the following equations:

(32) $$dD_{m}(\lambda )/d\lambda -dD_{\mathrm{refl}}\left(\lambda \right)/d\lambda =-bs\lambda^{-s-1},$$

(33) $$\log_{10}(-dD_{m}(\lambda )/d\lambda +dD_{\mathrm{refl}}\left(\lambda \right)/d\lambda )=-\left(s+1\right)\log_{10}\left(\lambda \right)+c.$$

Thus, in the case of a strong wavelength dependence of reflection losses, it is necessary to analyze the dependence of $\log_{10}(-dD_{m}(\lambda )/d\lambda +dD_{\mathrm{refl}}\left(\lambda \right)/d\lambda )$ on $\log_{10}\left(\lambda \right)$. This dependence may be compared with the dependence of $\log_{10}(-dD_{m}(\lambda )/d\lambda )$ on $\log_{10}\left(\lambda \right)$ which is considered in the new method (Equation (13)) and implies that the optical density of reflection losses is independent of wavelength.

**Figure 13:**
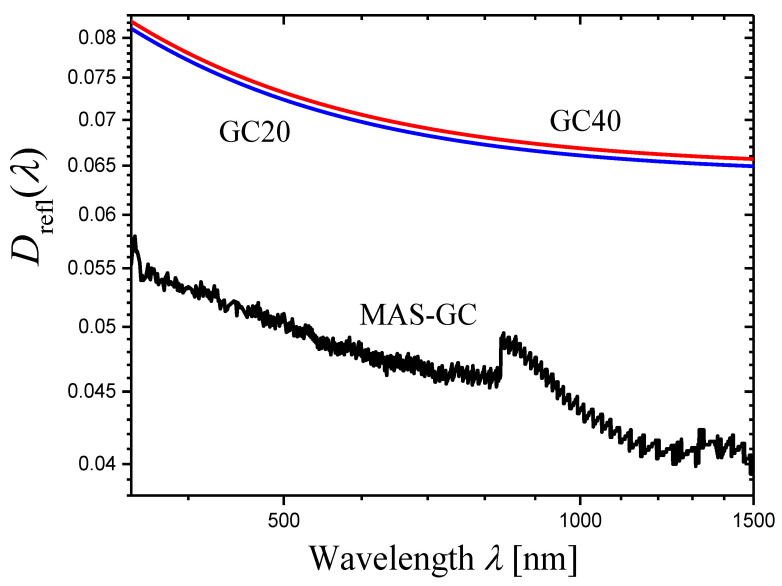
Optical densities of reflection losses, $D_{\mathrm{refl}}\left(\lambda \right)$, calculated for the glass-ceramics GC20, GC40 and MAS-GC (log–log plots). For the glass-ceramics GC20 and GC40, Equation (6) and analytical expressions for refractive index n(λ) were used. In the case of the glass-ceramic MAS-GC, calculation was carried out by Equation (34) using the measured optical densities of two samples.

Such analysis was carried out for the glass-ceramics GC20 and GC40 for which the dispersion of refractive index is large [41], and hence, there is the strong dependence of reflection losses on wavelength (Figure 13). To calculate $D_{\mathrm{refl}}\left(\lambda \right)$, we used analytical expressions for refractive index n(λ) [35] and Equation (6). The experimental data on $D_{m}\left(\lambda \right)$ were taken from Ref. [35] and used in the present paper earlier (Section 2.3 and this Section). Different values of step Δλ were used (Equation (30), Δλ≥5 nm). The exact values of the scattering exponent, $s_{\mathrm{exact}}$, were obtained using Equation (33) and compared with those estimated by the novel model assuming constant reflection losses (Equation (13)), s. The exact values are greater than the values estimated in the simple variant of the novel model, $s_{\mathrm{exact}}>s$; however, at all step values, the difference is insignificant ($s_{\mathrm{exact}}-s=0.033-0.037$ and 0.026−0.030 for GC20 and the GC40, respectively).

**Figure 14:**
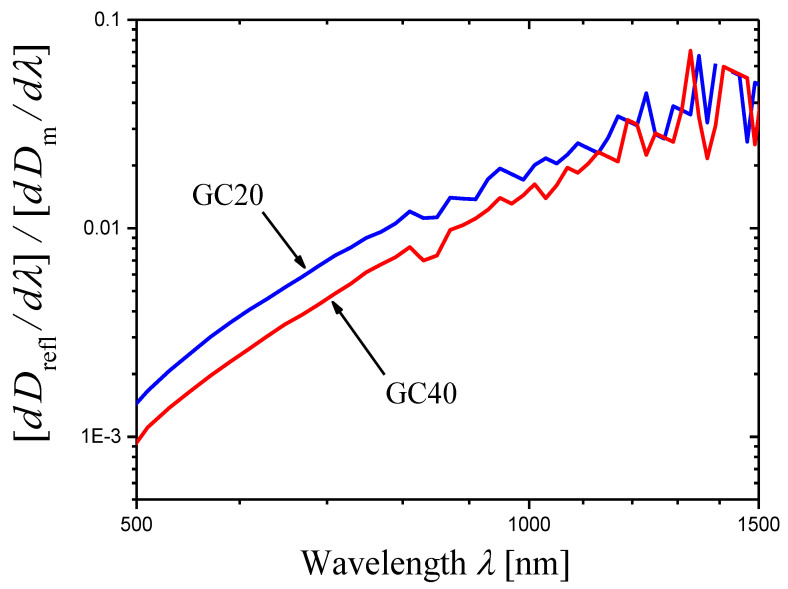
The ratio $\left[dD_{\mathrm{refl}}\left(\lambda \right)/d\lambda ]/[dD_{m}(\lambda )/d\lambda \right]$ for the glass-ceramics GC20 and GC40. The wavelength step is Δλ=20 nm.

In the case of GC20 or GC40, the curves of $\left[\log_{10}(-dD_{m}(\lambda )/d\lambda )]-[\log_{10}\left(\lambda \right)\right]$ and ${[\log}_{10}(-dD_{m}(\lambda )/d\lambda +dD_{\mathrm{refl}}\left(\lambda \right)/d\lambda )]-{[\log}_{10}\left(\lambda \right)]$ plots cannot be distinguished in the scale of Figure 11 at λ≲1000 nm. The reason is that the optical density of reflection losses changes with wavelength much more slowly than the optical density corresponding to light scattering. For illustration, we calculated the ratios $\left[dD_{\mathrm{refl}}\left(\lambda \right)/d\lambda ]/[dD_{m}(\lambda )/d\lambda \right]$ for GC20 and GC40 (Figure 14). Figure 14 shows that both ratios do not exceed 2% for wavelengths λ≲1000 nm. Thus, the curves plotted with and without consideration of the wavelength dependence of optical density of reflection losses practically coincide if the wavelength is not too long.

Let us consider another example in which the first method is used for determination of the internal optical density (or extinction coefficient) and the optical density of reflection losses depends on the wavelength. If the optical densities $D_{1m}\left(\lambda \right)$ and $D_{2m}(\lambda )$ measured for two samples with thicknesses $h_{1}$ and $h_{2}$, respectively, are known, the optical density of reflection losses can be found by the following equation:

(34) $$D_{\mathrm{refl}}\left(\lambda \right)=\left[h_{2}D_{1m}\left(\lambda \right)-h_{1}D_{2m}\left(\lambda \right)\right]/(h_{2}-h_{1}).$$

This equation follows from Equations (4) and (6). The dependence $D_{\mathrm{refl}}\left(\lambda \right)$ for MAS-GC (Section 2.2.4) calculated with the same data as for the construction of Figure 4a is presented in Figure 13. The step on the curve is due to the measuring procedure (switching between the detectors of the spectrophotometer). Anyway, we can state that $D_{\mathrm{refl}}\left(\lambda \right)$ for MAS-GC varies appreciably in the spectral range from 350 to 1100 nm, which is considered in Figure 5.

In this case, it is suitable to use the internal optical density D(λ) of a sample (see Equation (6)) and rewrite Equation (34) as:

(35) $$\log_{10}(-dD(\lambda )/d\lambda )=-\left(s+1\right)\log_{10}\left(\lambda \right)+c.$$

Thus, we may analyze the dependence of $\log_{10}(-dD(\lambda )/d\lambda )$ on $\log_{10}\left(\lambda \right)$, where D(λ) is internal optical density of a thin or a thick sample, and compare this dependence with the dependence of $\log_{10}(-dD_{m}(\lambda )/d\lambda )$ on $\log_{10}\left(\lambda \right)$ for the measured optical density $D_{m}(\lambda )$ of the same sample (the new method, Equation (13)). Using Equations (4) and (6), the internal optical density $D_{i}(\lambda )$ for each of two samples (i=1,2) can be expressed as:

(36) $$D_{i}\left(\lambda \right)=[h_{i}/(h_{2}-h_{1})][D_{2m}\left(\lambda \right)-D_{1m}\left(\lambda \right)].$$

**Figure 15:**
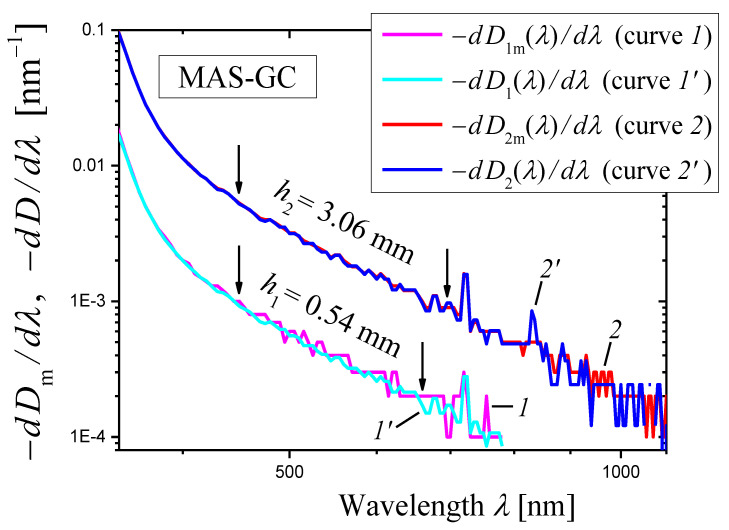
The plots of $\left[\log_{10}(-dD_{im}(\lambda )/d\lambda )]-[\log_{10}\left(\lambda \right)\right]$ (curves *1* and *2*) and ${[\log}_{10}(-dD_{i}(\lambda )/d\lambda )]-{[\log}_{10}\left(\lambda \right)]$ (curves *1*′ and *2*′) for the thin (i=1, curves *1* and *1*′) and thick (i=2, curves *2* and *2*′) samples of MAS-GC (Section 2.2.4).

Figure 15 shows the plots of $\left[\log_{10}(-dD_{im}(\lambda )/d\lambda )]-[\log_{10}\left(\lambda \right)\right]$ (curves *1* and *2*) and ${[\log}_{10}(-dD_{i}(\lambda )/d\lambda )]-{[\log}_{10}\left(\lambda \right)]$ (curves *1*′ and *2*′) for thin (i=1, curves *1* and *1*′) and thick (i=2, curves *2* and *2*′) samples of MAS-GC (Section 2.2.4). Curves *1* and *2* were obtained by the new method (Equation (13)) neglecting the wavelength dependence of the optical density of reflection losses (Equation (10)) and are also presented in Figure 5. The dependence of the optical density of reflection losses on wavelength was taken into account in plotting curves *1*′ and *2*′ (Equation (35)). One can see that the approximate curves (*1* and *2*) are close to the “exact” ones (*1*′ and *2*′) if oscillations are small (if the wavelength is not too long). The curves in Figure 15 demonstrate linear portions. In the ranges limited by arrows, each curve was approximated by a straight line using the least squares method. For curves *1* and *2*, the slopes $S_{\mathrm{lp}}$ are indicated in Figure 5. For curves *1*′ and *2*′, we had found the slopes $S_{\mathrm{lp}}=4.11\pm 0.13$ (*1*′) and 4.07±0.15 (*2*′) which are close to those given in Figure 5 for curves *1* and *2*. The corresponding “exact” values of the scattering exponent:

(37) $$s_{\mathrm{exact}}=3.11\pm 0.13\left(i=1\right),\;s_{\mathrm{exact}}=3.07\pm 0.15\left(i=2\right).$$

are very close to the values determined by the new method (Equation (22)).

Let us explain two features of curves *1*′ and *2*′ presented in Figure 15:

1. The internal optical densities of the thick and thin samples calculated by Equation (36) differ only by a constant factor: $D_{1}(\lambda )=(h_{1}/h_{2})D_{2}(\lambda )$. The similar equation also applies to derivatives: $dD_{1}(\lambda )/d\lambda =(h_{1}/h_{2})dD_{2}(\lambda )/d\lambda$. Thus, in the logarithmic representation (Figure 15), the curve of $dD_{1}(\lambda )/d\lambda$ (curve *1*′) can be obtained by shifting the curve of $dD_{2}(\lambda )/d\lambda$ (curve *2*′) along the y-axis. Figure 15 confirms this conclusion. A slight difference in slopes $S_{\mathrm{lp}}$ of curves *1*′ and *2*′ (see the text preceding Equation (37)) is due to different wavelength ranges of the linear approximation, shown by arrows in Figure 15.
2. If the thicknesses of the thin and thick samples are significantly different (as in the case shown in Figure 15), the internal optical densities $D_{1}(\lambda )$ and $D_{2}(\lambda )$ of both samples (see Equation (36)) are determined to a greater extent by the measured optical density of the thick sample, the relative measurement error for which is smaller than for the thin one. Therefore, the curve *1*′ is smoother than the curve *1*, obtained from measurements made for the thin sample.

To summarize, the considered examples of glass-ceramics GC20, GC40, and MAS-GC, characterized by a strong dependence of reflection losses on the wavelength, showed that the new method gives values of the scattering exponent, which practically do not differ from those obtained taking into account the spectral dependence of reflection losses.

Thus, the new method does not require investigation of the wavelength dependence of reflection losses and can be applied without restrictions.

## 4. Conclusions

Experimental data on the transparency of NGs (phase-separated glasses and glass-ceramics), presented in the literature and in this work, show that in a certain wavelength range, the scattering coefficient (turbidity) of these materials is frequently described by a power function of the inverse wavelength with an exponent which differs appreciably from the Rayleigh value 4 and is called the scattering exponent.

The determination of the scattering exponent is important for the optical applications of the material and for understanding the scattering mechanisms and the structure of the material.

Traditional methods for determining the scattering exponent are rather complex. The first (direct) method requires the preparation of two samples of different thicknesses. The second method requires information about the material’s refractive index for several wavelengths. To apply the third method, it is necessary to prove that the refractive index of the material does not change in the process of the formation of NG from the initial glass, and to have absorbance spectra of the initial glass and NG samples of the same thickness.

In this paper, a novel simple express method for the estimation of the scattering exponent is presented. In the method, the spectrum of the measured optical density for only one sample is used. The measured optical density is differentiated with respect to wavelength, and then the dependence of the derivative on wavelength is plotted in a log–log scale. The presence of a straight-line portion on this curve means that the scattering coefficient of the material is a power function of the inverse wavelength in a certain wavelength range with the exponent (the scattering exponent) which is less by unity than the absolute value of the slope of the straight-line portion.

The comparison showed that the scattering exponent values obtained by the new and three different previously developed methods are in good agreement. This conclusion was made based on a comparison of the scattering exponent values for NGs with amorphous and crystalline inhomogeneities of different nature, different sizes, and with different volume fractions of inhomogeneity regions.

The problem of the proper choice of the wavelength step and the sample thickness is discussed and the importance of this choice is noted.

Initially, it was assumed in the new method that the reflection losses do not depend on the wavelength. However, the consideration of examples, in which the strong dependence of reflection losses on the wavelength takes place, showed that the new method gives values of the scattering exponent which practically do not differ from those obtained taking into account the spectral dependence of reflection losses.

A conclusion was made that the new method does not require investigation of the wavelength dependence of reflection losses and can be applied without restrictions.

## Data Availability

The data presented in this study are available upon request from the corresponding author.
